# Biological effects of the frequent application of a copper-containing fungicide on the fruit fly Drosophila melanogaster

**DOI:** 10.21203/rs.3.rs-2556997/v1

**Published:** 2023-02-09

**Authors:** Daniele Zamberlan, Guilherme Rieder, Luis Silva, Joao Batista Teixeira da Rocha

**Affiliations:** Federal University of Santa Maria: Universidade Federal de Santa Maria; Federal University of Santa Maria: Universidade Federal de Santa Maria; Universidad de la Costa: Corporacion Universitaria de la Costa; Universidade Federal de Santa Maria Centro de Ciências Naturais e Exatas

**Keywords:** pesticides, environment, toxicity, behavior, memory retention

## Abstract

The increased consumption of pesticides has an environmental impact due to the dispersion of minerals. Bordasul^®^ is a commonly used fungicide composed of 20% Cu, 10% sulfur, and 3.0% calcium to correct its deficiency in plants. The evaluation of fungicide effects *in vivo* models is designed to assess their impact on the environment more broadly. Drosophila melanogaster offers a unique model due to its ease of handling and maintenance. Here, the effects of Bordasul^®^ were investigated, addressing the development, survival, and behavior of flies. Our findings showed that exposure to Bordasul^®^ prevented the development of flies (p < 0.01). In addition to causing a significant reduction in memory retention (p < 0.05) and locomotion capacity (p < 0.001). Although fungicides are necessary to satisfy the world’s food demand, we conclude that Bordasul^®^ is highly toxic, and that safer media, such as biofertilizers, must be developed as effective alternatives.

## 1. Introduction

Continuous growth in the human population worldwide has increased the demand for food production. The projection from the United Nations of 9 billion humans by 2050 translates to a doubling of the current food requirement (World population prospects, 2019). To meet these nutritional needs, the use of fertilizers and pesticides has become an indispensable tool in agriculture, contributing to huge improvements in food production.

Yet, the frequent application of these chemicals also has environmental impact effects (De Conti et al 2020). The large growing consumption of chemical fertilizers contributed to soil acidification (Schindler & Hecky 2009), water pollution (Rajput et al 2017), and increased atmospheric ammonia (Cancelier, 2016), methane (Bodelier, 2011) and carbon dioxide (Al-Kaisi et al 2008) emissions globally, due to dispersion of mineral use. However, there is scant information on the environmental impact of micronutrient pesticides, as they are commonly overlooked. The Bordeaux mixture, Ca(OH)_2_+CuSO_4_, (or Bordasul^®^ in Brazil), for example, is a commonly used fungicide composed of 20% Cu, 10% sulfur, and 3.0% calcium. It is also used as fertilizer to correct the deficiency of nutrients in plants. Considering that environmental Cu contamination is mainly derived from anthropogenic activities (Alemany et al 2017, Han et al 2014, Jan et al 2015, Safiur Rahman et al 2019, Williams et al 2019), this approach is of fundamental importance (Arora, 2018).

The evaluation of fungicide effects using *in vivo* models is a critical requirement to elucidate the broad impact of these inputs on the environment. *Drosophila melanogaster*, commonly known as the fruit fly, is an alternative model providing a bridge between *in vitro* and mammals and has been extensively used in research for more than 100 years. Its ease of manipulation and maintenance makes it an optimal model to study the environmental impact of fungicides on living organisms.

Herein, the effects of Bordasul powder were investigated in the fruit fly, focusing on its development, survival, and behavior.

## 2. Materials And Methods

### 2.1 Chemicals

The Bordeaux mixture, Ca(OH)_2_+CuSO_4_, (Bordasul^®^), a mixed mineral fungicide was commercially obtained.

### 2.2 *D. melanogaster* stock and culture

*D. melanogaster* (Harwich strain) used in the present investigation was obtained from the National Species Stock Center (Bowling Green, OH, USA). Diet was prepared as indicated by Adedara and collaborators (Adedara et al., 2015) with some adaptations. The flies were maintained and reared in corn meal medium (1% corn flour, 2% w/v brewer’s yeast, 2% w/v sucrose, 1% w/v powdered milk, 1% w/v agar, 0.08% w/v nipagin and 93.92% distilled water) at constant temperature (22–24°C) and relative humidity (60–70%) under 12-h dark/light cycle conditions.

For the experimental assays, adult flies (13 days-old) were anesthetized with ice between 5 to 10 minutes. Males and females were separated using a paintbrush and 10 females and 10 males were mated for 24 hours in 1% agar media (containing 0,5% defatted milk powder, 2% sucrose; 0,1% nipagin^®^, 1% yeast and different concentrations of Bordasul^®^ from 0; 0.1, 0.5 or 1 g/L of diet. The flies were removed, and the offspring used for the assays.

### 2.3 Exposure to Bordasul^®^

The offspring were randomly exposed to 3 different concentrations of the fungicide and the vehicle (water) as a control group. The highest exposure concentration was based on instructions provided in the product’s packaging (1 g/L). This was further diluted 1:2 representing the middle concentration, and 1:10 for the low concentration. Thus, the 3 concentrations used in ascending order were 0.1, 0.5, and 1.0 g/L low, medium, and high, respectively).

Given that in preliminary studies these concentrations affected fly development, we subsequently used concentrations 10 times lower for the behavioral assays, as follows: 0.01, 0.05, and 0.1 g/L.

The solutions were diluted in distilled autoclaved water, prepared at the time of use and mixed in a warm medium, and allowed to solidify and cool. All experiments were repeated 3 times and the number of flies used in each trial is contained in the description of the experiments.

### 2.4 Development assay

Fly development was measured by counting the survival rate at 5 days (larvae stage), 9 days (pupae stage), and 13 days (adult stage) after exposure to Bordasul or control (Zamberlan et al 2020a).

### 2.5 Lifespan assay

Lifespan assays were conducted as described above (Linford et al 2013), with some adaptations. After mating for 24 hours of the 10 pairs of flies, the offspring exposed to Bordasul^®^ concentrations from their oval phase was used for the lifespan assay. During the experimental period, flies were transferred to new vials containing fresh food 3 times a week in the absence of anesthesia to ensure that the feeding environment of young females was not interrupted by the presence of larvae. The longevity of the flies was counted every 2 days from the beginning of the adult phase.

Lifespan assays were conducted according to the previously described, with some adaptations. During the experimental period, flies were transferred onto new vials containing fresh food 3 times per week in the absence of anesthesia to ensure that the feeding environment for young females was not disrupted by the presence of larvae. The flies’ longevity was counted every 2 days from the onset of the adult stage.

### 2.6 Climbing assay

One the day before the climbing assay determination, flies were transferred to clean rearing flasks to avoid the interference of humidity in the quantification of flies’ climbing ability. For climbing assay, a vial (10 cm high × 1.5 cm of diameter) with a dotted line marked 6 cm away from the bottom was used. Groups of 10 flies were placed in the vial. The flies were tapped down to the bottom and allowed to climb. The number of flies that crossed the line marked in the vial within 6 seconds was recorded. Data were expressed as the percentage of flies that crossed the line within 6 seconds. The assay was repeated 3 times and the mean was calculated. At least 80 flies were tested per group (n equal or greater than 8 per group).

### 2.7 Memory retention assay

The effect of pairing an odorant to electrical shock was evaluated according to protocols previously described (Tully and Quinn, 1985) with adaptations described by Zamberlan et al., 2020. In the training session, the young (4 days-old) or aged (11 days-old) flies (30–50 flies from a given rearing flask) were exposed to a T maze (18 cm × 3 cm × 3 cm) in the presence of the odorant 3-octanol (OCT-1:100). Immediately after placing the flies in the maze, they received an electrical shock (75 V for 2 min, aversive pairing of OCT with shock). Then, the flies were exposed to methylcyclohexane (MCH-1:25) for 2 min without applying the shock. In the test sessions (which were performed 1, 30 or 60 minutes after the training session), the flies were again transferred to the T maze and allowed to choose for one of the arms of the maze (containing either the odor of MHC or OCT). But in these 3 test trials no shock was applied. The performance index was scored by recording the number of flies which chose the correct non-aversive arm (MCH) minus the number of flies that chose the wrong OCT arm divided by the total number of tested flies and presented as percentage.

### 2.8 Statistical analysis

All data are expressed as mean ± standard error of the mean (SEM). Statistical analysis was performed with GraphPad Prism7 Software. Survival and behavioral significance were assessed by one or two-way analysis of variance (ANOVA), followed by Newman-Keuls’s *post hoc* test. Survival curves were assessed by log-rank test for trend. Differences were considered statistically significant among groups at p < 0.05.

## 3. Results

### 3.1 Bordasul^®^ affects flies’ development

**Figure 1:**
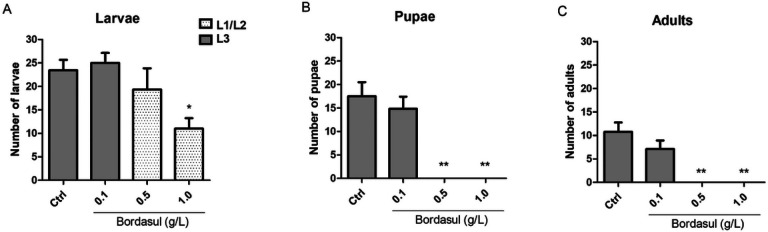
Effect of the fungicide Bordasul^®^ on *D. melanogaster* development. Bordasul in the highest concentrations significantly avoids flies development at (A) larvae (6 days after mating), (B) pulpae (12 days after mating), and (C) adult (18 days after mating) stage. *p<0.05 and **p<0.001 (compared to Ctrl group) one-way ANOVA followed by Bonferroni post-hoc test.

Exposure to Bordasul^®^ at 0.5 and 1.0 g/L led to an arrest in flies development (Fig. 1). A significant decrease in the number of larvae was observed in both Bordasul^®^ treatment groups when compared to the control group (Fig. 1. A - p < 0.05). Furthermore, flies treated with Bordasul^®^ failed to reach adult (Fig. 1. C), pupae (Fig. 1. B), and even the L3 stage (Fig. 1. A). Accordingly, in the following experiments, we used lower concentrations of Bordasul^®^ (as described in the methods) that did not affect their development.

### 3.2 Bordasul^®^ reduces flies’ longevity

**Figure 2:**
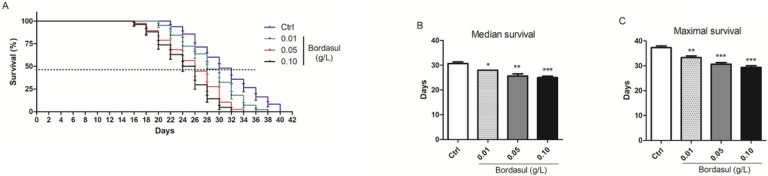
Effect of the fungicide Bordasul^®^ on *D. melanogaster* longevity. (A) Survival curves comparison p<0.001 Log-rank (Mantel-Cox) test. Bordasul^®^ significantly decreased flies’ (B) Median and (C) Maximal survival. *p<0.5; **p<0.01; ***p<0.001 (compared to the control group) one-way ANOVA followed by Bonferroni post-hoc test.

Exposure to Bordasul^®^ significantly affected *D. melanogaster* lifespan (p < 0.001 Log-rank Mantel-Cox test for curves comparison – Fig. 2.A). Bordasul^®^ led to a reduced median (50% alive) and maximal (90% alive) lifespan when compared to the control group (Fig. 2. B–C).

### 3.3 Bordasul^®^ decreases locomotor activity

**Figure 3:**
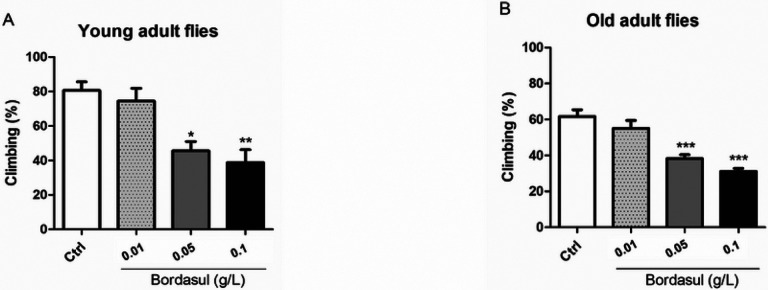
Effect of the fungicide Bordasul^®^ on *D. melanogaster*locomotor activity. The climbing assay was performed in the flies at (A) young-adult and (B) old-adult stage. *p<0.05; **p<0.01 and ***p<0.001 (compared to Ctrl group) one -way ANOVA followed by Bonferroni post-hoc test.

In the locomotor activity assay, flies exposed to Bordasul^®^ at 0.05 and 0.1 g/L showed a significant decrease in climbing at the young adult stage (p < 0.05), with a more pronounced effect at the higher Bordasul^®^ exposure (Fig. 3.A – p < 0.01). This effect was corroborated at an old-adult stage with an even greater effect (Fig. 3. B – p < 0.001).

### 3.4 Bordasul^®^ disrupts memory in flies

Memory retention test analysis indicated that the test was efficient in short-term memory acquisition since all flies avoided the odor paired with shock for 60 min after the shock, at both young (CI > 85% - Fig. 3. A) and old-adult (CI > 65% - Fig. 3. B) stages. No significant difference was observed between the groups in memory acquisition (time zero), except when comparing the young- to old-stage (data not shown). The analysis also demonstrated that flies exposed to 0.1 g/L of Bordasul^®^ had significantly lower memory retention at 60 min after memory acquisition at the young-adult stage when compared to control flies (Fig. 3. A – p < 0,05). This effect of treatment was more evident in old-adult flies, demonstrating defficient memory retention as early as 30 min after shock when compared to the control flies at the same age (Fig. 3. B – p < 0,05).

## 4. Discussion

Research has increasingly directed its focus toward safer and more efficacious agricultural products. The continuous increase in the consumption of fungicides and fertilizers in farming is necessary to meet global food demand, and as the population has grown, so has the utilization of these products. Concomitantly, their increased application has raised concerns about their environmental impacts. In this report, the *in vivo* toxicological effects of a chemical fungicide rich in copper (Cu) were demonstrated. We used the fruit fly *D. melanogaster* to investigate the biological effects of exposure to a fungicide commonly used in agriculture, namely, Bordasul^®^. It was used at environmentally-relevant concentrations per the manufacturer’s recommendations to evaluate its impact on the environment, herein, the fruit fly.

*D. melanogaster* is a well-established alternative and complementary model organism extensively studied for toxicity testing due to its inherent advantages over other more complex animal models (Yamaguchi & Yoshida 2018). *D. melanogaster* has a rapid life cycle and short lifespan, and large numbers of flies can be obtained in laboratory conditions (Reaume & Sokolowski 2006). It has been used as a model for the assessment of toxicological, developmental, as well as cognitive, memory, and learning deficit effects (Yamaguchi & Yoshida 2018, Zamberlan et al 2020a).

*D. melanogaster* behavior is susceptible to disruption by a broad spectrum of chemicals and environmental stresses. Behavioral changes in experimental models due to chemical or environmental stresses have been investigated in toxicity tests because it reflects the integrated physiological alteration (Chen et al 2015, Meyer & Williams 2014, Peres et al 2015, Tiwari et al 2011).

In this study, concentrations equal to or less than those recommended for use on the product’s label was tested in an attempt to mimic real-life exposure scenarios. Bordasul^®^ exposure in the larval period disrupted flies’ development at 0.5 mg/L, a concentration equivalent to half of what has been recommended for usage by the manufacturer. Furthermore, even lower concentrations, as low as 0.05 and 0.1 mg/L, led to a shorter lifespan, disrupted motor activity, and decreased memory retention at the adult stage when compared to the control. The effect on memory retention was more persistent in old flies compared to young flies, indicating the toxic effect on memory worsens over time.

The use of pesticides and chemical fertilizers has caused the degradation of soil quality and fertility mainly due to the accumulation of environmental pollutants. The chemical composition of Bordasul^®^ may be responsible for its toxic effects observed in *D. melanogaster*. Bordasul^®^ is rich in Cu, a trace element that plays important metabolic roles in all organisms (Bhattacharjee et al 2017, Sensi et al 2018, Vetchy), but can be toxic at altered levels, leading to memory and/or cognitive dysfunction (Kalita et al 2018, Sharma & Agrawal 2005) and accelerating diseases progression (Altarelli et al 2019, Bhattacharjee et al 2017, Mohr & Weiss 2019). It has been recently demonstrated that even modern nano-engineered Cu pesticides which have great promise for agricultural use given their intention of increasing effectiveness and reducing environmental harm, caused a similar magnitude of toxicity as conventional Cu pesticides (Vignardi et al 2020).

The fruit fly *D. melanogaster* has been widely used as an alternative model to study Cu metabolism and toxicity (Calap-Quintana et al 2017, Halmenschelager & da Rocha 2019, Navarro & Schneuwly 2017, Poulson et al 1952, Southon et al 2013). Drosophila cells express all classical genes involved in Cu regulation and their silencing altered Cu homeostasis, suggesting a conserved role. Previously, we demonstrated a toxic effect dependent on Cu concentration in *D. melanogaster* in relation to development, survival (Halmenschelager & da Rocha 2019), as well as decreased memory retention in pavlovian conditioning at the adult stage (Zamberlan et al 2020a, Zamberlan et al 2020b), where the concentrations of Cu used in these studies are close to those contained in the concentrations of Bordasul^®^ evaluated in the present study. Through this, we can infer that these concentrations of Cu in the chemical fertilizer may possibly be causing a reduction in survival, locomotor capacity and memory of the flies.

Cu imbalance has extensive effects on neural function and is significantly linked to cognitive deficits and neurodegenerative disease as Alzheimer’s pathology (Mao et al. 2012; Rembach et al. 2013; Squitti 2014). Schreurs 2013, showed that Cu retard learning in a rabbit model of Alzheimer’s disease (Schreurs 2013). Indeed, Cu exerts specific roles in the nervous system (Opazo et al 2014, Sellami et al 2012), which can be involved in the toxic effects exerted by Bordasul^®^.

These results are of fundamental importance in view of the constantly increasing demand for fungicides and fertilizers in the world. Agriculture is sustained with the help of chemical fungicides. Without them, it would not be possible to produce the great demand for food necessary to supply the world population. But along with these benefits comes environmental toxicity. The use of safer means, such as biofertilizers, can be an effective alternative to enable the production of necessary food in the world without affecting the environment.

## 5. Conclusion

The use of chemicals in agriculture contributes to environmental pollution, raising major concerns regarding ecological and ultimately human health effects. Tests to evaluate the biological effects of products used in the environment, mainly in food are of fundamental importance for the safety of both the environment and humans. Here, we demonstrated the toxic effects of the fungicide, Bordasul^®^, commonly used in farms, even in concentrations ten times lower than those indicated on its label. Overall, more extensive and systematic research efforts are required to investigate the effects of fertilizers on living organisms with a major focus on novel products.

## Figures and Tables

**Figure 4:**
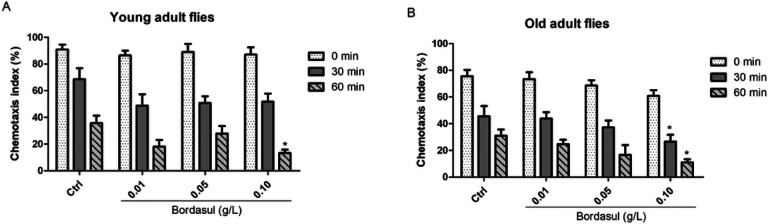
Effect of the fungicide Bordasul^®^ on *D. melanogaster* memory retention. The test was performed in (A) young (4 days old) and (B) old (11 days old) flies at the time 0, 30, and 60 min after the shock. 0.10 g/L Bordasul-exposed old adult flies presented decreased memory retention at all time points when compared to the Ctrl group. *p<0.05 (compared to Ctrl group) one-way ANOVA followed by Bonferroni post-hoc test.
